# Antibiotic prophylaxis in stone surgery: a systematic review of the literature

**DOI:** 10.1007/s00345-025-05528-1

**Published:** 2025-03-03

**Authors:** Filippo Gavi, Mauro Ragonese, Daniele Fettucciari, Maria Chiara Sighinolfi, Filippo Turri, Enrico Panio, Stefano Moretto, Giovanni Balocchi, Domenico Maria Sanesi, Filippo Marino, Alessandra Francocci, Pierluigi Russo, Nazario Foschi, Francesco Pinto, Emilio Sacco, Bernardo Rocco

**Affiliations:** 1 Department of Urology, Fondazione Policlinico Universitario A. Gemelli IRCCS, Rome, Italy; 2 Università Cattolica del Sacro Cuore, Roma, Italia; 3 Department of Urology, Ospedale Fatebenefratelli Isola Tiberina, Gemelli Isola, Roma, Italy; 4 Department of Urology, Humanitas Clinical and Research Institute IRCCS, Rozzano, Milan, Italy; 5 Department of Urology, Humanitas Gavazzeni, Bergamo, Italy

**Keywords:** Antibiotic prophylaxis, Stone surgery, Endourology, Percutaneous nephrolithotripsy, Urolithiasis, Stone culture

## Abstract

**Purpose:**

Endoscopic lithotripsy has become widely adopted worldwide and is considered the gold standard for managing upper urinary tract lithiasis. One of its possible complications is post-operative infection. We aimed to review available literature about the role of preoperative antibiotic prophylaxis and its indications.

**Methods:**

We performed a systematic search of the literature including “ureteroscopy”, “PNL”, “retrograde intrarenal surgery”, “antibiotic prophylaxis” and “sepsis” as keywords. Out of 760 relevant studies only 13 met our inclusion criteria: at least 10 adult patients (≥ 18 years old) diagnosed with kidney or ureteral stones; antibiotic prophylaxis described and reported; post-operative sepsis, UTI or SIRS assessed.

**Conclusions:**

Antibiotic prophylaxis strategies for stone surgery show different degrees of effectiveness depending on both the approach and the patient’s condition. Today’s evidence suggests that while routine antibiotic prophylaxis might not be necessary for all patients with sterile urine undergoing ureterorenoscopy and lithotripsy vs. percutaneous nephrolithotripsy, for those patients with positive preoperative urine cultures it is beneficial reducing the risk of postoperative infective complications.

## Introduction

Urolithiasis is a prevalent condition affecting between 1% and 20% of the global population, with variations depending on geographical regions [1]. Over the past five decades, there has been a global significant increase in the number of cases, with its incidence rising by 48.6% between 1990 and 2019 [2]. This period has also seen significant advancements in minimally invasive techniques for treating urolithiasis, including ureteroscopy (URS), shock wave lithotripsy (SWL), retrograde intrarenal surgery (RIRS), and percutaneous nephrolithotomy (PNL) [3]. RIRS has become a widely adopted procedure across urological departments, emerging as a principal surgical method for managing upper urinary tract stones [4, 5]. However, RIRS is not without risks; complications can include ureteral perforation, ureteral stricture, renal function loss, and postoperative infections [3, 6]. Among these, infections pose a particular concern, ranging from postoperative fever to potentially life-threatening urosepsis [6], especially in the context of nosocomial infections [7]. Recent systematic review and meta-analysis report postoperative sepsis rates following RIRS ranging from 0.2 to 17.8% [8], consistent with findings from newer studies showing sepsis rates between 0.5% and 11.1% [9].

For large renal stones, PNL remains the standard treatment [10]. A systematic review involving nearly 12,000 patients reported complications associated with PNL, with fever occurring in 10.8% and sepsis in 0.5% of cases [11]. According to EAU guidelines, antibiotic prophylaxis is recommended to lower the risk of symptomatic urinary infections following URS (strength rating: weak). Single-dose antibiotic prophylaxis is advised for PNL to reduce clinical urinary infections (strength rating: strong). In contrast, prophylactic antibiotics are not recommended for reducing infection rates in SWL [10]. We aim to address these infection risks and evaluate current antibiotic regimens by conducting a systematic review of the effectiveness of preoperative antibiotic prophylaxis in preventing postoperative urinary tract infections (UTIs).

## Materials and methods

### Search strategy

The study protocol was registered in in the International Prospective Register of Ongoing Systematic Reviews (PROSPERO registration ID: CRD42024493766). This systematic review was conducted according to the Cochrane review guidelines and following the Preferred Reporting Items for Systematic Reviews and Meta-analyses (PRISMA) checklist [12]. A systematic literature search was performed in February 2024 using PubMed and Scopus databases. No time nor language restrictions were applied. The search terms included “ureteroscopy”, “PNL”, “retrograde intrarenal surgery”, “antibiotic prophylaxis” and “sepsis”. The entire string is available in the supplementary materials. Duplicated studies from the same author’s group were excluded, retaining the ones fulfilling the selection criteria and the most recent ones; however, if they fulfilled the selection criteria and provided additional information on outcomes of interest, they were retained for these outcomes only.

### Study selection and eligibility criteria

Studies were considered for inclusion if they (1) included at least 10 adult patients (≥ 18 years old) diagnosed with kidney or ureteral stones, (2) antibiotic prophylaxis for RIRS, URS, PNL or endoscopic combined intrarenal surgery (ECIRS) was described and reported, (3) post-operative sepsis, UTI or systemic inflammatory response syndrome (SIRS) was assessed. RCTs and prospective studies comparing antibiotic prophylaxis during stone surgery with a placebo, standard of care or no antibiotic prophylaxis were included. Reviews, commentaries, authors’ replies, editorial letters and case reports were excluded. The population, interventions, comparison, outcomes, and eligibility criteria for the meta- analysis are shown in Table 1. After retrieving the articles from all the selected databases, duplicates were removed, and the initial screening by title and abstract was performed using the website tool Rayyan [13], which permitted independent screening of the articles by the researchers according to the triple-blind methodology to reduce selection bias [14].

**Table 1:** Study design, endpoints and eligibility criteria

Population (*P*)	Adults (≥ 18 years old) diagnosed with kidney or ureteral stones undergoing RIRS, URS, PNL, or ECIRS.
**Intervention (I)**	Antibiotic prophylaxis for stone surgery.
**Comparison (C)**	Placebo, standard of care, or no antibiotic prophylaxis.
**Outcome (O)**	Incidence of post-operative sepsis, urinary tract infection (UTI), or systemic inflammatory response syndrome (SIRS).
**Study Design**	Randomized controlled trials (RCTs) and prospective studies.
**Exclusions**	Reviews, commentaries, authors’ replies, editorial letters, and case reports.

Notes: RIRS: Retrograde Intrarenal Surgery; URS: Ureteroscopy; PNL: Percutaneous Nephrolithotomy; ECIRS: Endoscopic Combined Intrarenal Surgery

### Data extraction

The studies were independently reviewed by three authors (D.F.; F.G. and D.S.) based on the inclusion criteria, and key data on study characteristics and outcomes were collected using a standardized form [15]. If any data were missing, the authors were contacted via email to request additional information.

### Study quality assessment

Two authors (D.S. and D.F.) independently evaluated included studies’ quality. The risk of bias of the included studies was assessed with the Cochrane Collaboration’s tool for assessing the risk of bias [16]. Disagreements were resolved via consultation with a third co-author (M.C.S.).

## Results

### Study selection and characteristics

**Fig. 1:**
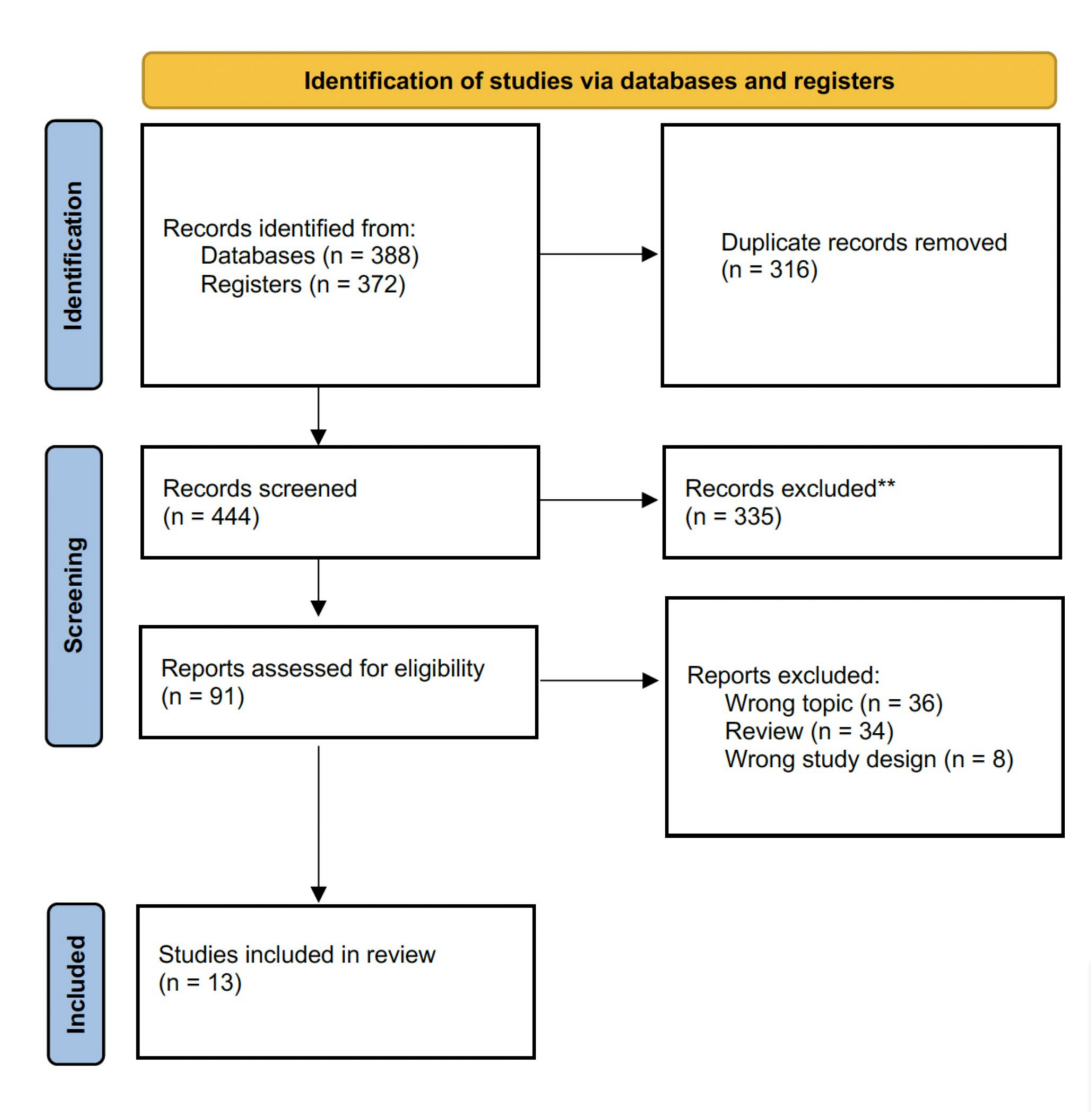
PRISMA flowchart for study selection

We summarized the study selection process in the PRISMA flowchart (Fig. 1). A total of 760 relevant studies were identified. Of these, 91 studies full-text articles were assessed for eligibility, and 13 studies met our inclusion criteria [17–29]. Baseline characteristics of the included studies are shown in Tables 2 and 3. Six of the studies were RCTs [18–23]. Seven studies were prospective studies [17, 24–29]. Seven t studies assessed antibiotic prophylaxis in ULT/URS [18–24, 27]. Six of the studies assessed antibiotic prophylaxis in PNL [17, 25, 26, 28, 29].

**Table 2:** Included studies on ureteroscopy (FURS: flexible ureteroscopy)

Author	Study type	Country	Patients	Inclusion criteria	Intervention/control	Outcome parameter	Outcome	Remarks
**El-Agami et al. (2023)**	RCT (B)	Saudi Arabia; Egipt	*N* = 256 (128/128)	FURS No symptomatic UTI.	2 g ceftriaxone i.v. 48 h prior untill 24-hour post-operative (p.o.); 500 mg ciprofloxacin i.v. one hour before surgery and 500 mg ciprofloxacin p.o. 24-hour post-operative.	UTI and sepsis	ABP with ciprofloxacin gives: Significative decrease in UTI 6.3% -> 15.6% (*p* = 0.04)	Hospital readmission was mandatory in 10 cases (3.9%) for UTI and urosepsis.
**Hsieh et al. (2014)**	RCT (A)	Taiwan	*N* = 212 (53/53/53/53)	≥ 18 y/o. FURS. No risk factors (pyruria, positive leukocyte esterase, AB 4 wk prior, immunedepression). Allergy to quinolone or cephalosporines.	Single dose 500 mg levofloxacin p.o.; Single dose 1 g cefazolin i.v.; Single dose 1 g ceftriaxone i.v.; Placebo.	Fever, UTI	ABP significantly decreases postoperative pyuria 48.4% vs. 64.7% (*p* = 0.04)	Proximal stones were at a higher risk of developing postoperative fUTI than those with middle and distal ureteric stones (6.5 vs. 0.7%, odds ratio [OR] 9.35, 95% CI 1.07–82.0; *p* = 0.03)
**Knopf et al. (2003)**	RCT (B)	Germany	*N* = 113 (57/56)	FURS No risk factors (clinical signs of infections, leukocytosis, AB 1 wk prior, pregnancy, allergy to AB)	Single dose 500 mg levofloxacin i.v.; No prophylaxis.	UTI	ABP with levofloxacin gives: Significant decrease in bacteriuria 12.5% vs. 1.8% (*p* = 0.026)	Nosocomial induced bacteriuria was reduced with ATP 10.7% vs. 0% (*p* = 0.011)
**Kobayashi et al. (2021)**	Prospective	Japan	*N* = 162	FURS	2 g cefazolin i.v. 24-hour prior surgery; (+) urine culture pre-operative AB 3/7 days depending on antibiogram and AB i.v. 24-hour prior	Fever, qSOFA, sepsis	Fever 13 (21%) pts; sepsis 1 (1%) pts	Patients with (+) urine culture pre-operative had higher complications rate 16% vs. 2%, *p* = 0.001
**Li et al. (2020)**	RCT (A)	Taiwan	*N* = 109 (36/37/36)	FURS Age 20–75 y/o. (-) urine culture pre-operative	Single dose 1 g cefazolin i.v.; Single dose 500 mg cefuroxime p.o.; No prophilactic antibiotics	Fever	No differences in fever rates among the 3 groups. Cefazoline 3 (9.4%); Cefuroxime 2 (6.9%); No ABP 0; *p* > 0.05	Comparison of prophylactic antibiotics in patients without pyuria undergoing ureterorenoscopic surgery
**Quiao et al. (2018)**	RCT (A)	China	*N* = 216 (101/105)	Retrograde semi-rigid ureteroscopic lithotripsy	Two doses 3 g fosfomycin tromethamine p.o.; Other antibiotics based on local standards	UTI, fever, sepsis	No differences between the two groups. UTI following lithotripsy was 3% vs. 6.1% (*p* > 0.05)	
**Gokalp et al. (2023)**	Prospective	Turkey	*N* = 286 (49/137)	FURS	3 g Fosfomycin tromethamine 4–6 h before surgery; Cephalosporin 30 min before surgery + additional dose 6 h after surgery	UTI, fever, sepsis	No statistical difference between the two groups for fever and UTI (*p* = 0.408 and *p* = 0.438, respectively)	Preoperative UTI and previous ESWL were independent risk factors for p.o. infectious complications

**Table 3:** Included studies on PCNL (BT: body temperature; HR: heart rate; WBC: white blood cells; NR: not reported; Pts: patients; UC: urine colture; RPUC: renal pelvis urine cultures; SC: stone culture; FT: fosfomycin Tromethamine)

Author	Study type	Country	Patients	Inclusion criteria	Intervention/control	Outcome parameter	Outcome	Remarks
**Fourcade et al. (1990)**	RCT	France	*N* = 120	≥ 18 y/o and ≤ 60 y/o Undergoing stone surgery (PCNL or endoscopic lithotripsy)	Single dose 1 g Cefotaxime before surgery; Placebo	SIRS or sepsis	SIRS in 53 (16.7%) pts	Preoperative positive UCs, intraoperative positive RPUCs, and SCs are strongly correlated with the development of SIRS (*p* = 0.001)
**Korets et al. (2011)**	Prospective	USA	*N* = 198	Patients undergoing PCNL	(-) pre-operative urine culture: broad spectrum antibiotics before surgery and in p.o. day 1; (+) pre-operative u.c.: at least 7 days treatment based on antibiogram. No control	SIRS or sepsis; BT ≤ 36ºC or ≥ 38ºC; HR > 100 bpm; > 20 breaths per minute; 12,000 wbc/µl or ≤ 4000 wbc/µl.	SIRS in 20 (9.8%) pts	SIRS was more frequent in patients with struvite calculi, longer operative time, positive pelvic and stone cultures.
**Mariappan et al. (2006)**	Prospective	UK	*N* = 98 (52/46)	(-) pre-operative urine culture. Dilated pelvicalyceal systems and/or stones of ≥ 20 mm	1-week course of ciprofloxacin before PCNL. Vs no supplemental ATP All patients received 5 mg/kg gentamicin i.v. at the time of induction for the PCNL.	SIRS or sepsis	ABP with 7 days ciprofloxacin gives: - Significative postoperative decrease in SIRS (RR, 3.4, 95% CI 1.0–11.8, *P* = 0.04)	Pelvic urine resulted three times less infected than control group
**Omar et al. (2019)**	Prospective	Egypt	*N* = 84 (41/43)	(-) pre-operative urine culture or a culture specific treatment of infection. - No AB for 1 wk prior.	Single dose 200 mg ciprofloxacin i.v. Two doses 1 mg cefotaxime i.v.	Postoperative fever, stone culture, SIRS, sepsis	ABP with ciprofloxacin gives significative postoperative decrease in fever 28% -> 5% (*p* = 0.02)	No differences in fever, SIRS or sepsis is (-) or (+) urine preoperative
**Song et al. (2019)**	Prospective	China	*N* = 112 (53/59)	(-) pre-operative urine culture or a culture specific treatment of infection. Patients undergoing PCNL	3 gr FT p.o.; 3 gr cefuroxime i.v.	Postoperative SOFA score, fever > 38ºC, PCT	ABP with FT reduces the colony count and increases ATB concentration in the stones (*p* < 0.05; *p* < 0.05)	SOFA score was significantly lower in the experiment group
**Walton-Diaz et al. (2016)**	Prospective	Chile Argentina	*N* = 122	Patients undergoing PCNL	(-) pre-operative urine culture: p.o. single dose 3rd generation cephalosporin; (+) pre-operative u.c.: at least 7 days treatment based on antibiogram.	Sepsis	PMUC results were different than RPUC and RSC	They recommend running RPUC and RSC in patients at high risk of developing infectious complications, to guide treatment

### Risk of bias

**Fig. 2:**
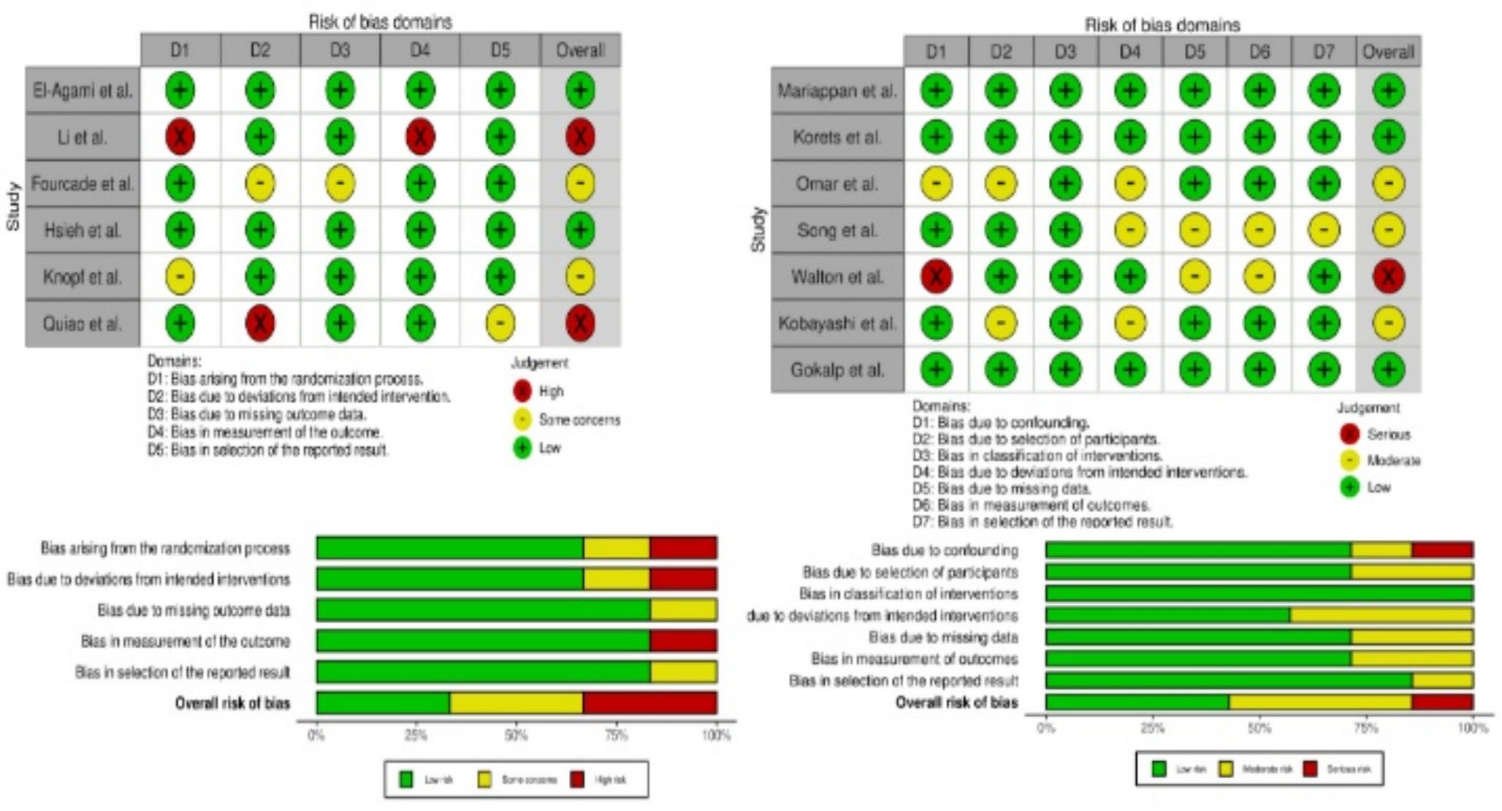
Risk of bias assessment

We summarized RoB assessment in Fig. 2. Five studies were judged to have low risk of bias [18, 21, 24, 26, 28], five studies at moderate risk of bias [20, 22, 25, 27, 29] and three at high risk of bias [17, 19, 23].

### Targeted antibiotic prophylaxis based on preoperative urine culture

Walton et al. [17] conducted a prospective multicenter study to evaluate the concordance of microbiological profiles from preoperative midstream urine culture (PMUC), renal pelvis urine culture (RPUC) and renal stone culture (RSC). One hundred and twenty-two patients who underwent PCNL were enrolled. All patients were tested with PMUC: if negative they underwent a single-prophylactic dose of a third-generation cephalosporin during anesthetic induction; 4 patients (3.2%) had positive PMUC and received specific antibiotic treatment for at least 7 days prior to surgery or continued antibiotic treatment until a negative PMUC was obtained. Isolated agents were multidrug-susceptible E. Coli and S. Aureus. After surgery, patients were monitored for symptoms and signs of sepsis, which was confirmed with a new urine culture, two aerobic hemocultures and a thorax X-Ray to rule out pulmonary causes. Eighteen (14%) patients had a positive RPUC while seventeen (13,9%) had a positive RSC. Results from PMUC were different to those obtained from RPUC, which showed multidrug resistant bacteria and fungus. Considering this, they concluded standard recommended antibiotic prophylaxis would not cover these agents. Therefore, they recommend running RPUC and RSC, especially in patients at high risk of developing infectious complications to guide the eventual treatment. El-Agami et al. [18] conducted a prospective RCT comparing the rate of post-flexible ureteroscopy UTI in patients subjected to the standard antibiotic prophylaxis alone versus enhanced prophylactic measures. The study included 256 patients undergoing flexible ureteroscopy (FURS) for ureteral or renal stones. Patients in Group 1 received an intravenous injection of fluoroquinolone one hour before surgery, and oral antibiotics were used for 24 h postoperatively. Patients in Group 2 (enhanced prophylaxis) were tested with a urine culture ten days before the procedure; antibiotic-culture based was given for positive asymptomatic cases, while the procedure was deferred for active UTI. Postoperative, and overall complications were significantly higher in group 1 (15.6% vs. 6.3%, *p* = 0.04 and 26.6% vs. 17.2%, *p* = 0.047), respectively. Twenty patients (15.6%) in the standard prophylaxis group were diagnosed with UTI in comparison to 8 patients (6.3%) in the enhanced prophylaxis group (*p* = 0.047). Therefore, authors concluded that UTI after FURS could be reduced significantly by utilizing the suggested enhanced prophylactic approach.

### Antibiotic prophylaxis vs. no prophylaxis in patients with sterile urine culture

Li et al. [19] conducted a prospective open-label RCT enrolling 109 patients undergoing RIRS. Patients were randomly assigned to one of three groups, receiving one of the following three treatments: a single dose of intravenous cefazolin (1 g), oral Cefuroxime (500 mg), and no prophylactic antibiotics. After surgery, no postoperative antibiotics were given unless febrile UTI was diagnosed. Febrile UTI was lower in the cefuroxime group (0%) than in the cefazolin group (9.4%, *n* = 3), but this difference was not statistically significant (*p* = 0.1). No statistical significance was found between the prophylaxis group and the no-prophylaxis group. In conclusion, as antibiotic prophylaxis could not reduce the incidence of postoperative febrile UTI and pyuria in patients with sterile urine undergoing RIRS, they suggested not giving antibiotic prophylaxis to this population to avoid antibiotic resistance and adverse events. Fourcade et al. [20] conducted an RCT to determine the importance of antibiotic prophylaxis before stone surgery (percutaneous or endoscopic) in patients with a preoperatory sterile urine culture. A total of 120 patients were divided into two groups: one received a single cefotaxime 1 g iv at induction of anesthesia, and the other received a placebo. Patient follow-up was divided into two periods: from day 1 to 3 and from day 3 to 30. The incidence of postoperative bacteriuria between the first and third postoperative days was significantly higher in the placebo group than in the cefotaxime-treated group (*p* = 0.014). The chemical stone type did not influence the outcome of postoperative infection. During the day 3 to day 30 follow-up period, no infection symptoms occurred in the cefotaxime group, while seven infectious episodes were noted in the placebo group (of which 6 UTIs). With an early postoperative infection rate of 23% in the placebo group as against 8.3% in the cefotaxime treated group (*p* = 0.014), this controlled study’s results favored antibiotic prophylaxis. The study by Hsieh et al. [21] aimed to determine the efficacy of prophylactic antibiotics in reducing post-surgical infections in patients undergoing URS. They conducted a double-blind, prospective, randomized controlled trial, enrolling 206 patients with preoperative sterile urine undergoing URSL. Patients were randomly allocated, in a ratio of 1:1:1:1, to receive prophylactic antibiotics with a single dose i.v. Cefazolin (1 g), Ceftriaxone (1 g), or oral Levofloxacin (500 mg), or no treatment (control group), respectively. Urinalysis and urine cultures were obtained between postoperative days 5 and 7. The rates of postoperative pyuria were significantly lower in patients with prophylaxis than in the control group (48.4 vs. 64.7%, *P* = 0.04). Patients receiving prophylaxis with levofloxacin and ceftriaxone had a significantly lower risk of pyuria than the control group (52.0 and 36.5 vs. 64.7%, respectively; *P* < 0.05). The rates of bacteriuria and fUTI tended to be lower in patients with prophylaxis, although the difference was not significant (4.5 vs. 11.8%, *P* = 0.09, 1.3 vs. 5.9%, *P* = 0.09). There was no significant difference in rates of bacteriuria and fUTI between the four groups. Patients with proximal stones had a higher risk of developing postoperative fUTI (odds ratio 9.35; *P* = 0.03). The authors concluded that antibiotic prophylaxis significantly reduces the incidence of pyuria after URSL and tends to diminish the risk of bacteriuria and UTI. Knopf et al. [22] conducted a study to determine whether perioperative single-shot prophylaxis in connection with a URS stone removal influences the rate of postoperative UTI. One hundred and thirteen patients were included in this prospective randomized study. In 57 patients, 250 mg Levofloxacin p.o. was given approximately 60 min before ureteroscopy, and 56 patients had no prophylaxis. In the group without prophylaxis, the rate of postoperative significant bacteriuria was significantly higher than in the group with prophylaxis (seven patients [12.5%] vs. one patient [1.8%]) (*p* = 0.026). In six cases, there was an *E. coli* bacteriuria. Additionally, a *K. Pneumoniae* and a non-specified *Staphylococcus* bacteriuria were detected in other cases. In conclusion, the authors suggest a single-shot prophylaxis using 250 mg Levofloxacin p.o. can be considered a valuable prophylaxis option. In addition, perioperative single-shot prophylaxis may be beneficial in case of an unexpected intraoperative complication, e.g., ureter perforations.

### Antibiotic prophylaxis: fosfomycin tromethamine vs. standard of care

The primary endpoint of Qiao et al. [30] prospective, multicenter, RCT was to define the incidence of infectious complications (defined as asymptomatic and symptomatic bacteriuria, fever, urosepsis, bacteremia, and genitourinary infection) in patients undergoing RIRS. They enrolled 2016 patients and randomly divided them into two groups: the first received fosfomycin tromethamine, while the control groups received antibiotics according to local standard of practice (second-generation cephalosporin, fluoroquinolones, and other intravenous antibiotics). The overall incidence of infections following lithotripsy was 4.6%, with no statistically significant difference between study groups. Authors concluded fosfomycin is a valid cost-effective alternative to cephalosporin and fluoroquinolones for prophylactic purposes. Gokalp et al. [24] conducted a non-randomized controlled study enrolling 186 patients undergoing RIRS. Preoperative urine cultures were collected from all patients before surgery and, if positive, the intervention was postponed. Patients were divided into two groups: Group 1 (*n* = 49) received an oral dose of 3 g of Fosfomycin tromethamine administered 4–6 h before surgery; Group 2 received intravenous 1 g third-generation Cephalosporin (ceftriaxone) 30 min before surgery and an additional dose 6 h after surgery. The authors found no statistically relevant difference between the groups in terms of postoperative fever and UTI (*p* = 0.408 and *p* = 0.438, respectively) and suggested the use of Fosfomycin tromethamine as preoperative prophylaxis, even in patients with a positive preoperative urine culture.

### Antibiotic prophylaxis: ciprofloxacin vs. cefotaxime

Omar et al. [25] conducted a prospective study in patients with preoperatory sterile urine culture before PCNL. Between February and October 2016, 84 patients were enrolled. These patients were divided into two groups: 1°) 41 patients were given a single dose of 200 mg ciprofloxacin infusion, and 2°) 43 patients were given 2 mg of cefotaxime divided into two doses. Two (5%) patients in the ciprofloxacin group developed fever compared to 12 (28%) patients in the cefotaxime group. Twelve patients in the ciprofloxacin group (29%) and 14 in the cefotaxime group (33%) had a positive stone culture. RPCU was positive in 7 patients in the ciprofloxacin group and 10 patients in the cefotaxime group (17% vs. 23%). None developed clinical septic shock. In the ciprofloxacin and cefotaxime groups, SIRS 4% vs. 7% and fever 1% vs. 5% were identified, respectively. A prophylactic regimen consisting of a single dose of ciprofloxacin infusion during surgery induction showed a higher efficacy as a preoperative antibacterial preparation. Korets et al. [26] conducted a prospective study to define the correlation between the development of SIRS and preoperative UC, intraoperative RPUC and SC in patients undergoing PCNL. From February 2009 to February 2011, 198 patients were enrolled. All patients with a negative UC received a preoperative dose of prophylactic intravenous broad-spectrum antibiotics, which were continued for 24 h postoperatively. If the UC was positive, it received a minimum 7-day course of oral antibiotics based on the sensitivity profile. Broad-spectrum intravenous antibiotics were used at induction. At induction was administered broad-spectrum intravenous antibiotics. UC was positive in 47 cases (23.5%); RPUC in 21 (10.3%); SC in 33 (16.2%). SIRS was noted in 20 cases (9.8%); 6 of the 20 patients (30%) required intensive care treatment. Compared to patients without a postoperative-SIRS, those with SIRS were more commonly female (75% vs. 46%), had longer operative time (146 min vs. 113 min), had a higher rate of positive pelvic (26% vs. 10%) and stones (44% vs. 17%) cultures. Even appropriate treatment of preoperative urinary infections may not prevent a postoperative systemic response after PNL but it seems to result in a decreased rate of bacteremia and possibly hastened recovery from SIRS. Kobayashi et al. [27] aim to study the influence of preoperative antimicrobial treatment on intraoperative culture (IC) results and infectious complications in patients with positive preoperative bladder urine culture (PBUC) undergoing ureteroscopic lithotripsy. They enrolled 162 patients from April 2019 to March 2020. Based on PBUC findings, they were divided into positive and negative PBUC groups. Preoperative antimicrobial treatment was administered to the positive PBUC group while the negative PBUC group received perioperative antimicrobial prophylaxis only. IC, including bladder urine culture, renal pelvic urine culture, and stone culture, were collected. In the positive PBUC group, 19 (28.4%) patients had positive bladder urine cultures after the antibiotic treatment. Positive ICs (43.3% vs. 3.2%, *p* < 0.001) and post-operative fever (16.4% vs. 2.0%, *p* = 0.001) were more common in the positive PBUC group than in the negative PBUC group. In the positive PBUC group, 11 patients had a postoperative fever, regardless of the ICs results (6 positive ICs and 5 negative ICs). Furthermore, antimicrobial-resistant bacteria were detected from ICs in 5 patients with positive PBUC, including 4 suffering from a post-operative fever. Although the effect of pre-operative antimicrobial treatment is not definitive, to avoid serious infectious complications, authors concluded that careful infection control, based on the pre- and intra-operative culture results, should be performed at least in high-risk patients. Mariappan et al. [28] conducted a prospective study to evaluate whether 1 week of ciprofloxacin before surgery reduces SIRS in patients with stones > 2 cm or with pelvi-calyceal dilatation undergoing PNL. All the 98 patients included had negative urine culture before PNL. All patients received iv gentamycin 5 mg/kg at induction. In the ciprofloxacin-treated group there was a significant reduction of SIRS (7% vs. 18%) with an incidence three times less of infected pelvic urine. Song et al. [29] conducted a prospective study to evaluate the antibacterial effect of Fosfomycin tromethamine (FT) on the bacteria inside urinary infection stones in patients undergoing PNL. A total of 112 patients with a negative urine culture were examined from May to December 2018. The patients were divided into two groups: the experimental group received oral FT 3 g/day, and the control group received intravenous infusion of cefuroxime 3 g/day. The measured drug concentrations inside the stones suggest that the drug concentration within the stones in the experimental group was significantly higher than in the control group. The colony count in the stone cultures from the experimental group was also lower than the control group. There was no significant difference between the two groups in postoperative fever risk. Moreover, the SOFA scores showed that the risk of systemic organ failure was significantly lower in the experimental group (SOFA > 2 control group 33.26% vs. experimental group 10%).

## Conclusions

Antibiotic prophylaxis strategies for stone surgery show varying degrees of effectiveness depending on the approach and the patient’s condition. The evidence suggests that, while a single dose peri-operative antibiotic prophylaxis might be enough for all patients with sterile urine undergoing renal stone surgeries, targeted and culture-based antibiotic strategies are beneficial, especially in high-risk patients with positive preoperative cultures. Enhanced prophylactic measures, including preoperative urine cultures and tailored antibiotics, significantly reduces postoperative infection rates and complications. Additionally, nowadays antibiotic resistance represents a global health issue, amplified by the overuse of broad-spectrum antibiotics. Tailored therapies, based on preoperative UC and local resistance patterns, ensure a higher efficacy of target antibiotics, minimizing at the same time unnecessary exposure to broad-spectrum agents. Stone culture is a promising diagnostic and preventive tool although it’s still missing clinical evidence in reducing infectious complications.

Limitations of this study lie in the heterogeneity of the works included (RCTs and prospective studies) and, because of the moderate to high risk of bias present in several of the studies, the reliability of their findings might be affected. Furthermore, the different definitions of outcomes, like postoperative infections, might affect the consistency of the review.

## Data Availability

No datasets were generated or analysed during the current study.
